# Evaluating the ability of a trauma team activation tool to identify severe injury: a multicentre cohort study

**DOI:** 10.1186/s13049-018-0533-y

**Published:** 2018-08-10

**Authors:** Ole-Petter Vinjevoll, Oddvar Uleberg, Elaine Cole

**Affiliations:** 1 Department of Surgery, St. Olav’s University Hospital, 7006 Trondheim, Norway; 2 Department of Emergency Medicine and Pre-Hospital Services, St. Olav’s University Hospital, 7006 Trondheim, Norway; 3 Department of Research and Development, Norwegian Air Ambulance Foundation, Drøbak, Norway; 4 Centre for Trauma Sciences, The Blizard Institute, Bart’s and the London School of Medicine and Dentistry, Queen Mary, University of London, London, UK

**Keywords:** Trauma, Injury, Triage, Overtriage, Undertriage, Trauma team activation

## Abstract

**Background:**

Sensitive decision making tools should assist prehospital personnel in the triage of injured patients, identifying those who require immediate lifesaving interventions and safely reducing unnecessary under- and overtriage. In 2014 a new trauma team activation (TTA) tool was implemented in Central Norway. The overall objective of this study was to evaluate the ability of the new TTA tool to identify severe injury.

**Methods:**

This was a multi-center observational cohort study with retrospective data analysis. All patients received by trauma teams at seven hospitals in Central Norway between 01.01.2015 to 31.12.2015 were included. Severe injury was defined as Injury Severity Score (ISS) > 15. Overtriage was defined as the rate of patients with TTA and ISS < 15, whilst patients with TTA and ISS > 15 were defined as correctly triaged.

**Results:**

A total of 1141 patients were identified, of which 998 were eligible for triage criteria analysis. Median age was 35 years (IQR 20–58) and the male proportion was 67%. Mechanism of injury was predominantly blunt trauma (96%) with transport related accidents (62%) followed by falls (22%) the most common. Overall, median injury severity score (ISS) was low and severely injured patients (ISS > 15) comprised 13% of the cohort. Utility of specific TTA criteria were: physiology 20%, anatomical injury 21%, mechanism of injury (MOI) 53% and special causes 6%. Overtriage among all patients was 87%, and for those with physiologic criteria 66%, anatomical injury 82%, mechanism of injury 97% and special causes criteria 92%, respectively.

**Conclusions:**

Severe injury was infrequent and there was a substantial rate of overtriage. The ability of the TTA tool was relatively insensitive in identifying severe injury, but showed increased performance when utilizing physiologic and anatomical injury criteria. Many of the TTA mechanism of injury criteria might be considered for removal from the triage tool due to substantial rates of overtriage. This has relevance for the proposed development of national Norwegian TTA criteria.

## Background

To optimize outcomes the severely injured patient should be taken to the correct facility with immediate assessment by a trauma team upon arrival to the hospital [1, 2]. In order to determine the requirement for trauma team activation (TTA) on arrival to hospital, it is important to identify the severely injured patient in prehospital care. Sensitive decision making tools should assist prehospital personnel in the triage of injured patients, safely identifying those who require TTA. Failure to recognise severe injury, known as undertriage has been found to cause delayed diagnosis and therapeutic interventions, and is associated with missed injuries and increased morbidity [2, 3]. Conversely overtriage might result in unnecessary use of available hospital resources by alerting the trauma team for those with minor injury [4].

TTA tools include criteria which indicate physiologic derangement, anatomical injuries and mechanism of injury (MOI) likely to cause severe injury [5]. Altered physiologic and anatomic criteria are reported to best predict severe injury, whereas MOI has been found to show a low precision in predicting severe injury, resulting in substantial over triage [3, 6–10]. In 2014 the Central Norway Trauma System (CNTS) established a new tool for TTA to minimize variation in prehospital triage decisions. This tool was subsequently adopted by all hospitals in the region (Table 1). The intention of the new tool was to introduce the same set of criteria to all pre-hospital emergency medical services (EMS), regional emergency medical communication centres (EMCC) and local hospitals, to provide a consistent tool for recognition of the severely injured patient within the region. However two recent publications reported that the majority of published trauma triage tools lack the sensitivity and specificity to predict severe injury [3, 11]. Comparing the field triage criteria implemented in CTNS with to those recommended by Centers for Disease Control and Prevention (CDC) [12], twelve out of thirty criteria were either lacking or defined differently (Table 1). The discriminatory ability of the new TTA criteria in use within Central Norway was unknown therefore the efficacy of the CNTS tool required investigation.

**Table 1:** Trauma team activation criteria

CRITERIA	1. PHYSIOLOGY
1.1	In need of ventilation support (bag-mask, intubated/attempted intubation)
1.2	Respiration Rate < 10/min
1.3	Respiration Rate > 29/min (adults)
1.4	Saturation (SpO_2_) < 90% without supplemental oxygen^a^
1.5	Pulse Rate > 130/min (in combination with other physiological/anatomical variables)^a^
1.6	Systolic blood pressure < 90 mmHg
1.7	GCS ≤ 13 (if significant head injury consider directly to MTC)
1.8	Severe hypothermia (< 30 ° C)(when in cardiac arrest consider direct transport to MTC)^a^
	2. ANATOMICAL INJURY
2.1	Facial injury with potential airway compromise^a^
2.2	Unstable chest wall (flail chest/multiple rib fractures)
2.3	Severe external bleeding^a^
2.4	Amputation proximal to ankle/wrist (consider conference with Oslo University Hospital)
2.5	Penetrating injury proximal to knee/elbow
2.6	Severe crush injury
2.7	Bilateral femur fracture
2.8	Severe pelvic pain^a^
2.9	Suspected spinal cord injury (neurological deficit)
	3. MECHANISM OF INJURY
3.1	Car crash > 70 km/h with seatbelt or triggered airbag^a^ or Car crash > 50 km/ without seatbelt or no triggered airbag^a^
3.2	Vehicle rollover^a^
3.3	Entrapment with compartment intrusion^a^
3.4	Ejection from vehicle
3.5	Falls from > 5 m (adults) or > 3 m (children)
3.6	Motorcycle crash > 30 km/h
3.7	Pedestrian/bicyclist struck by vehicle
3.8	Death in same passenger compartment
	4. SPECIAL CAUSES
4.1	Age > 60 years^a^
4.2	Age < 5 years^a^
4.3	Severe co-morbidity^a^
4.4	Pregnant /gestational age > 20 weeks
4.5	Knowledge of active use of anticoagulants or known bleeding disorder

^a^criteria different from the 2011 field triage criteria by Centers for Disease Control and Prevention (CDC) [12]

The overall objective of this study was to evaluate the ability of the new TTA tool to identify severe injury. Specifically the primary aim was to determine which triage criteria are more precise in identifying severe injury. Secondly, to identify if there are any criteria that can safely be removed from the TTA to simplify the process for clinicians and avoid unnecessary use of hospital resources.

## Methods

This was a multi-center observational cohort study with retrospective data analysis.

### Clinical setting

Central Norway is one of four major health trusts in Norway. It covers an area of 56.385 km^2^ and a total population of 680.110. St. Olav’s University Hospital is the major trauma centre (MTC) and has formal responsibility for the regional trauma organization [13, 14]. Injured patients in need of multidisciplinary intensive care or those in the need of special surgical treatment (neuro- and cardiothoracic surgery) are admitted directly or transferred from acute care hospitals (ACH). Four hospitals are designated as ACH with trauma receiving capability and two additional hospitals has no trauma receiving capacity, but are included in the study as local patients continued to be admitted there before transfer to a MTC or ACH. Trauma team activation is performed by EMCC specially trained nurses according to the TTA tool (Table 1).

### Study population

All patients in the seven hospitals that had been met by a trauma-team upon admission between 01.01.2015 to 31.12.2015 were included. This time frame was chosen to allow time (eight months) for the TTA tool to initially embed. Deaths prior to hospital arrival, patients without TTA and those transferred from other hospitals more than 24 h after injury were excluded.

### Data collection

Data including age, gender, mechanism of injury, on-scene physiologic values and prehospital intubation were collected prospectively upon hospital arrival. Additional data including emergency surgical procedures were collected from in-hospital electronic patient records, EMCC information system and EMS reports. Data was extracted retrospectively for study purposes. Injury Severity Score (ISS) was used to define injury severity using the Abbreviated Injury Scale (AIS) – Revision 2005 and Injury Severity Score (ISS) > 15 was considered severe injury [15–17].

### Data analysis

Descriptive characteristics of the study sample are presented as medians with inter-quartile ranges (IQR), or absolute numbers with percentages and 95% confidence intervals (CI). Primary outcome was rate of overtriage within specific criteria groups. Based on prehospital information, the criterion used for activation of the TTA tool was determined for each patient. If several criteria were utilized, the criterion with the worst anticipated clinical impact was selected in ascending order of severity with physiology (most severe) to special causes (least severe). We used overtriage rates to assess the ability of TTA criteria to identify severe injury. Overtriage was defined as the rate of patients with TTA and ISS < 15, whilst patients with TTA and ISS > 15 were defined as correctly triaged. Data analysis was performed using SPSS statistical software (IBM Corporation, released 2015. SPSS Statistics for Windows, Version 22, IBM Corporation, Armonk, NY, USA).

## Results

During the study period, 1141 injured patients were received by trauma teams within the region. A total of 143 patients had no criteria to dispatch the TTA, therefore this left 998 patients for analysis. The cohort was predominantly male (67%), at a median age of 35 years (IQR 20–58). The principal mechanisms of injuries were transport related incidents (62%) and falls (22%). Overall the severity of injury was low (median ISS 4, IQR 1–9) with severely injured patients (ISS > 15) comprising only 13% of the cohort. The severely injured patients were older, required more prehospital intubations, increased transfusions and a higher rate of emergency procedures (Table 2). Seventeen patients (2%) died within 30 days after the incident of which 71% had suffered severe injuries.

**Table 2:** Baseline characteristics of included patients

	All patients	Severe Injury (ISS > 15)
Total *n (%)*	998 (100)	127 (100)
Age in years *median (IQR)*	35 (20–58)	43 (25–66)
Male *n (%)*	668 (67)	100 (78)
Blunt trauma *n (%)*	955 (96)	119 (93)
Transport related accidents *n (%)*	663 (66)	63 (50)
Falls *n (%)*	196 (20)	39 (30)
Other *n (%)*	70 (7)	14 (11)
Stabbed by sharp object *n (%)*	35 (4)	4 (3)
Struck or hit by blunt object *n (%)*	32 (3)	7 (6)
Shot by gun of any dimension *n (%)*	2 (< 1)	0
Systolic blood pressure *median (IQR)*	132 (120–148)	120 (112–150)
Systolic blood pressure < 90 mmHg *n (%)*	25 (3)	10 (8)
Glasgow Coma Scale *median (IQR)*	15 (15–15)	14 (10–15)
Glasgow Coma Scale ≤8 *n (%)*	58 (6)	30 (23)
Respiratory rate per min > 30 or < 10 *n (%)*	49 (5)	16 (13)
Patients intubated pre-hospital *n (%)*	36 (4)	24 (19)
Patients receiving transfusions *n (%)*	20 (2)	13 (10)
Patients receiving thoracic drainage *n (%)*	39 (4)	28 (22)
Emergency surgery procedures *n (%)*	35 (3)	26 (20)
- Damage control thoracotomy *n (%)*	2 (0.2)	2 (0.2)
- Damage control laparotomy *n (%)*	18 (2)	11 (9)
- Limb revascularization *n (%)*	1 (0.1)	0
- Craniotomy *n (%)*	7 (0.7)	6 (5)
- Intracranial pressure device insertion *n (%)*	7 (0.7)	7 (5)
Injury Severity Score (ISS) *median (IQR)*	4 (1–9)	22 (17–29)
30 day Mortality *n (%)*	17 (2)	12 (9)

Overtriage amongst all patients included was 87%, and for those with physiologic criteria 66%, anatomical injury 82%, mechanism of injury 97% and special causes criteria 92%, respectively (Table 3). There were variations amongst individual TTA criteria and the ability to identify severely injured patients. The physiology criteria – Glasgow Coma Scale (GCS) ≤ 13, ‘in need of ventilation support’ and the anatomical injury criteria - severe pelvic pain and unstable chest wall, were most associated with a higher rate of severe injury than other criteria in the same groups (Table 4). All physiologic criteria except pulse rate above 130/min and severe hypothermia were present in the TTA initiation of severely injured patients. MOI criterion performed poorly at predicting severe injury, specifically vehicle-roll over, falls, vehicle entrapment and ejection from vehicle (Table 4).

**Table 3:** Performance of trauma team activation criteria when an Injury Severity Score (ISS) > 15 defines severe injury

	N (%)	ISS > 15 N (%)	Overtriage % (95% CI)
Patients with TTA criteria (all)	998 (100)	127 (100)	87 (87–88)
Criteria - physiology	197 (20)	67 (52)	66 (62–70)
Criteria – anatomical injury	214 (21)	39 (31)	82 (77–85)
Criteria - mechanism of injury	524 (53)	16 (13)	97 (95–98)
Criteria - special causes	63 (6)	5 (4)	92 (87–96)

*CI* confidence interval, *TTA* trauma team activation

**Table 4:** Trauma team activation by single criterion prevalence

Criterion	All patients	Severe Injury (ISS > 15)
Physiology		
GCS ≤ 13	81 (8)	24 (19)
Respiration rate > 29/ min (adults)	40 (4)	7 (6)
In need of ventilation support	37 (4)	26 (20)
Saturation (SpO_2_) < 90% without supplemental oxygen ^a^	17 (2)	6 (5)
Systolic blood pressure < 90 mmHg	9 (1)	2 (2)
Respiration rate < 10/min	8 (1)	2 (2)
Pulse rate > 130/min ^a^	5 (0.5)	0
Severe hypothermia ^a^	0	0
Anatomical injury		
Severe pelvic pain ^a^	72 (7)	12 (9)
Unstable chest wall	70 (7)	13 (10)
Penetrating injury proximal to knee/elbow	29 (3)	3 (2)
Suspected spinal cord injury	18 (2)	4 (3)
Facial injury with potential airway compromise ^a^	11 (1)	4 (3)
Severe crush injury	8 (1)	1 (1)
Severe external bleeding ^a^	6 (1)	2 (2)
Amputation proximal to ankle/wrist	0	0
Bilateral femur fracture	0	0
Mechanism of injury		
Car crash ^a^	291 (29)	6 (5)
Motorcycle crash > 30 km/h	99 (10)	3 (2)
Pedestrian/bicyclist struck by vehicle	47 (5)	2 (2)
Death in same passenger compartment	42 (4)	5 (3)
Vehicle rollover ^a^	33 (2)	0
Falls	7 (1)	0
Entrapment with compartment intrusion ^a^	3 (0.3)	0
Ejection from vehicle	2 (0.2)	0
Special causes		
Age > 60 years ^a^	48 (5)	5 (3)
Age < 5 years ^a^	11 (1)	0
Pregnant / gestational age > 20 weeks	3 (0.3)	0
Severe co-morbidity ^a^	1 (0.1)	0
Use of anticoagulants or known bleeding disorder	0	0

^a^criteria different from the 2011 field triage criteria by Centers for Disease Control and Prevention (CDC) [12]. Data are presented as n (%)

## Discussion

In this first multicenter study after implementation of a new TTA tool within the region of Central Norway, severe injury was infrequent within the trauma population and there was a substantial rate of overtriage. The performance of the TTA tool was relatively insensitive in identifying severe injury, but showed increased precision in physiology and anatomical injury criteria. Many of the TTA mechanism of injury criteria might be considered for removal from the triage tool due to substantial rates of overtriage.

Identifying the severely injured patient in the prehospital environment is challenging. The setting may be chaotic, with little clinical information available, together with the need for emergent intervention and the requirement for rapid extrication to the most appropriate health facility. Therefore criteria have been developed to ease the identification process based on physiology, anatomical injury and mechanism of injury conditions [5, 12, 18, 19]. The initial goal of the criteria, named “field triage decision process”, was to ensure that patients were conveyed to a trauma center or hospital best equipped to manage the specific injuries, but has gradually been adopted for use as in-hospital TTA criteria [12, 20]. Within six of the CTNS criteria different from CDC recommendations (pulse rate > 130/min, severe hypothermia, vehicle roll-over, entrapment with compartment intrusion, age < 5 years and severe co-morbidity), no patients with severe injuries were identified (Table 4). Ideal criteria should be both 100% sensitive (identifying all patients with severe trauma) and specific, but a feasible measure is suggested which might allow over triage rates up to 50% in order to minimize under triage [5]. In this study there was a low caseload of severely injured patients and the high rate of overtriage is anecdotally accepted on the assumption that TTA provides valuable training for the trauma system. Overtriage has most often been described as a human and economic resource problem, but the overuse of TTA has also been associated with an increased incidence of adverse events for non-trauma emergency patients when concurrently admitted [20–23]. This underlines the need to increase precision of TTA criteria in reducing the negative impact of other patient groups requiring contemporaneous resources [21].

In our study we based the ability to recognize severe injury defined by an ISS > 15. In a recently published systematic review the definition of under- and overtriage relied on the ability to transport severely injured patients to a Level 1 trauma center [3]. Virtually all of the protocols had a low sensitivity, thereby failing to identify severe injury and this was complicated by a lack of consensus for what constituted severe injuries [3]. Varying definitions may therefore lead to different interpretations on measures of precision. As seen in our study, several patients with ISS < 15 initially had an indication of severe injury with GCS ≤ 8 (*n* = 28), systolic blood pressure < 90 mmHg (*n* = 15), pre-hospital intubation (*n* = 12) and requiring emergency surgery (*n* = 9). According to our definition these were not subsequently defined as severely injured. In a study by Palmer et al. investigating pediatric patients, the use of a lower threshold of an ISS of 8 or higher was considered a better definition of a “severely injured” patient, if morbidity, mortality and the need of resources is to be considered [24]. This requires further evaluation in adult patients, especially for the elderly, where ISS < 15 may not reflect the true burden of injury.

In our study physiologic and anatomic criteria generally showed lower rates of overtriage than MOI, consistent with other studies [25, 26]. Historic studies highlighting the use of MOI criteria originate from the 1990s, however there has been considerable development in automotive and safety technology during the last decades, suggesting that the use of single MOI criteria in activating the trauma team should be reconsidered [5, 27]. Falls from any height comprised 22% of the cohort, yet altered physiologic or anatomic variables were the better predictors of severe injury than the MOI itself. Recent evidence from the UK suggests that falls are an increasing cause of severe injury in adults [28], and that a simple MOI such as a low level fall may not initially present as major trauma. This may indicate the need for an increased focus on attaining and monitoring trends in vital signs at an early stage to evaluate physiologic response to a seemingly ‘minor’ injury. MOI criteria only predicted 3% of severe injury and this emphasizes the low discriminatory ability of using MOI as single criteria. Yet this study suggests that some of the vehicle related MOI criteria, namely car crash, motorcycle crash, pedestrian struck by vehicle and death in the same passenger compartment should remain in the TTA protocol given their ability to predict ISS > 15. Based on our findings, larger studies are required to determine if other MOI criteria may be considered for removal without the threat of failing to identify severely injured patients. These criteria in the MOI sections of the protocol included rollover, entrapment and ejection, and whilst they were found in forty-five patients triage decisions, none were subsequently characterized as severe injury. In 2011 Sasser et al. decided to not include rollover as a stand-alone criterion in their guidelines because of less than 20% PPV for ISS > 15, which was the threshold for inclusion as a criterion in MOI [12]. The same panel also chose to exclude extrication of entrapped patients from the guidelines due to insufficient evidence to support the ability to detect those severely injured. Entrapment with compartment intrusion and vehicle roll-over were implemented as criteria in CNTS, though no patients with these criteria were severely injured. However the findings of our study would need to be validated in a larger data set, with interim recommendations to not use MOI as single criteria for TTA decisions.

In this present study, 13% of patients had severe injury, in which four specific criteria appeared to be the most predictive in the majority of these cases. These findings support previous evidence where reduced GCS was associated with increased risk of severe injury and prehospital advanced airway techniques were reported the strongest predictor of death or increased length of stay in hospital [29]. In a recommendation by the Centers for Disease Control and Prevention (CDC) in 2011, “need for ventilator support” was added because of these findings [12]. Anatomical injury criteria showed a high precision in identifying severe injury, which fits with the recommendation for TTA in patients with “chest wall instability or deformity” or “pelvic fractures” [12]. Age above 60 years was the only special cause criteria which correlated with severe injury. The rates of elderly trauma are increasing globally and low energy mechanisms are associated with severe injury in this cohort [28, 30]. Reasons for increased mortality in geriatric trauma are attributed to pre-existing diseases mask physiologic response to injury (e.g. beta-blockers), and thus affect triage decision making [3, 31]. In this region, age-related special cause criteria remains an important component of the current TTA protocol.

This study has some limitations. First, using retrospective data is dependent on several registrars accurately inputting the initial data, which may affect the level and consistency of documentation. Second, due to the retrospective design and regional data capturing systems, there was no possibility to register all trauma admissions without TTA and those patients admitted with minor injuries not in need of TTA. This led to the inability to assess sensitivity, specificity, positive and negative predictive values of TTA criteria groups and single criterion. Finally, the study focused solely on patients with severe injury (ISS > 15), yet those patients with moderate injuries (ISS 9–15) may require TTA and this requires further prospective national evaluation.

## Conclusions

Severe injury was infrequent within the trauma population and there was a substantial rate of overtriage. The ability of the TTA tool was relatively insensitive in identifying severe injury, but showed increased performance when utilizing physiologic and anatomical injury criteria. Several TTA initiated by mechanism of injury criteria might be considered for removal from the triage tool due to substantial rates of overtriage. This has relevance for the proposed development of national Norwegian TTA criteria.

## Abbreviations

ACH: Acute Care Hospital
AIS: Abbreviated Injury Scale
CDC: Centers for Disease Control and Prevention
CI: Confidence Interval
CNTS: Central Norway Trauma System
EMCC: Emergency medical coordination centre
EMS: Emergency Medical Services
GCS: Glasgow Coma Scale
IQR: Interquartile Range
ISS: Injury Severity Score
MOI: Mechanism of Injury
MTC: Major trauma Centre
TTA: Trauma team activation
UK: United Kingdom
